# Acceptability of self-administered antigen test for COVID-19 in the Philippines

**DOI:** 10.1017/S0266462324000035

**Published:** 2024-01-17

**Authors:** Jayne Eunice U. Yang, Faisal H. Jackarain, Tisha Isabelle M. de Vergara, Joshua F. Santillan, Patrick Wincy C. Reyes, Ma Cecilia Victoria B. Arellano, Jainor Timothy U. Garcia, Sheena Jasley G. Samonte, Anne Julienne G. Marfori, Anna Melissa S. Guerrero

**Affiliations:** Department of Health, Manila, Philippines

**Keywords:** biomedical, COVID-19 serological testing, health impact assessment, self-administration, self-testing, technology assessment

## Abstract

**Objectives:**

In response to the Omicron surge in early 2022, the HTA Philippines evaluated the acceptability of Filipinos in using self-administered antigen tests (SAAgTs) as part of COVID-19 HTAs in the Philippines.

**Methods:**

Scoping review from literature databases was initially conducted to identify preset codes in the use of SAAgT. Preset codes were used to establish the questions for focus group discussions (FGDs). Semi-structured questionnaires were created through Delphi technique. FGDs with four stakeholder groups (i.e., nine healthcare workers [HCWs], seven representatives of at-risk groups, six economic frontliners, and seven representatives of micro–small–medium-sized enterprises) were conducted.

**Results:**

Discomfort in being a target of stigma and being prescribed an “illness identity” when suspected or confirmed COVID-19-positive, along with lack of confidence to perform self-test, caused hesitancy in self-testing among participants. The need for subsidies for test kits from the government or employers was emphasized to increase its accessibility. Having a designated access point and reporting system for SAAgT was highlighted to avoid nepotism (*padrino* system attributed to debt of gratitude), inequitable distribution, and lapses in reporting. A participatory approach to education was perceived as crucial to reduce any misconceptions associated with the use of SAAgT.

**Conclusions:**

All FGD groups expressed favorable reviews on the implementation of SAAgT because it can potentially reduce the burden of health facility-administered tests. These findings were considered by the HTA Council in the recommendation of SAAgT as part of the overarching national strategies for the diagnosis and screening of COVID-19.

## Introduction

### Burden of disease

During the COVID-19 pandemic Omicron surge in March 2022, the national health systems and various economic and social sectors were incapacitated all over the globe. According to the World Bank, emerging and developing economies are the most heavily affected with regard to the overutilization of national resources to mitigate pandemic spread, minimize economic losses as a result of lockdowns, and pacify citizen unrest [1]. The Philippines, as a developing economy, did not have adequate capacities to efficiently deal with the burden of the disease. Resources are unequally distributed throughout the country, being more concentrated in only urbanized regions [2]. The average ratio of human resources for health (HRH) in the country—19.7 health workers to 10,000 population—does not follow the World Health Organization benchmark of 44.5 health workers per 10,000 population. This gap widens during pandemic surges [3;4].

The ratio mismatch between HRH capacity and the volume of patients directly impacts the health facilities’ efficiency to deliver COVID-19 testing services.

### COVID-19 testing in the Philippines

Reverse transcription polymerase chain reaction (RT-PCR) remains the reference test in the Philippines. Trained professionals are required in order to accurately perform the diagnostic test. Moreover, it has a long turnaround time (3 to 5 days) due to the referral process between institutions or local government units (LGUs) and respective hospital epidemiological surveillance units. Most testing centers are located in commercialized areas, while some provinces lack laboratories that provide RT-PCR, especially geographically isolated and disadvantaged areas (GIDAs). Thus, the surge in COVID-19 cases alongside increasing demand for RT-PCR testing caused delays in diagnosis and release of test results, especially in regions that lack basic health facilities [5].

Apart from long turnaround time, many Filipinos are not enthusiastic about getting a COVID-19 diagnostic test in the health facility. Most Filipinos perceive the RT-PCR to be costly, even with the price cap set by the Department of Health (DOH) [9]. Perceived high cost and slow release of RT-PCR results fuel the unregulated selling of antigen test kits in the market, many of which are overpriced [6].

These challenges called for a need to introduce a testing strategy that is cheaper, readily accessible, easy to use, and has a faster turnaround time for those who need immediate test results. The rapid antigen test (RAgT) was introduced to the country in 2021 as a cheaper alternative to the RT-PCR with much faster turnaround time [7]. Subsequently, the Philippine Food and Drug Administration issued a special certification for the use of self-administered antigen tests (SAAgTs) in 2022 as a “test which can be performed by non-healthcare professionals or lay users in home, nonhospital, or nonlaboratory settings.” SAAgTs are administered similarly as RAgTs but target nasal/midturbinate or saliva instead of nasopharyngeal/oropharyngeal specimen so that self-administration without much discomfort can be feasible for the general public [8].

### Purpose of the assessment

Pursuant to the role of the Health Technology Assessment (HTA) Council in the Universal Health Care Act of 2019 in providing recommendations on financing coverage decisions of health technologies including COVID-19 test kits, this assessment was conducted to explore the acceptability of SAAgT among potential users in the Philippines. This acceptability assessment is part of an overarching HTA on the clinical benefit and cost-effectiveness of SAAgT in order to further support its recommendation for government financing by the DOH. This can allow government financing for SAAgT which can potentially increase testing capacity in the country and subsequently mitigate future case surges. The assessment was guided by the following research questions:

- What are the perceptions of using SAAgTs among Filipinos?
- What is the level of acceptability in using SAAgT among Filipinos?
- How can the self-reporting capability and willingness in using SAAgT be described?

## Methods

**Figure 1.**
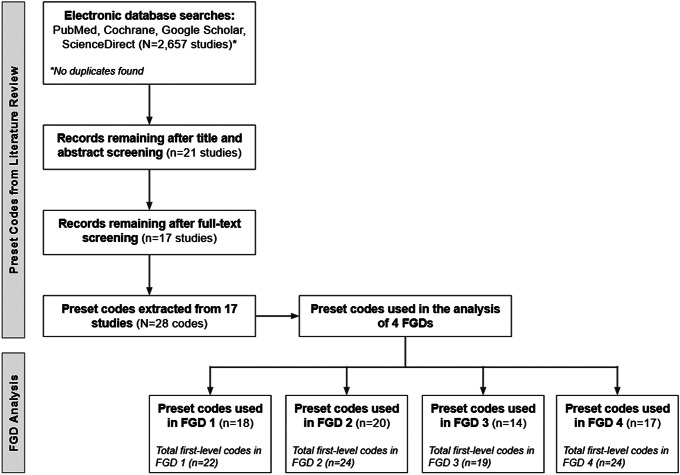
Methodology diagram.

Two main approaches were used in order to collect data about the acceptability of Filipinos in using SAAgT (see Figure 1). The first approach, literature scoping, was conducted in order to identify key themes about self-testing in existing literature. The second approach is the conduct of focus group discussions (FGDs) through the use of a semi-structured questionnaire. The instrument used in the FGDs was created using Delphi Technique, in which experts from the HTA Council were consulted during its conceptualization. These two approaches are detailed below.

### Literature scoping

- ***Data collection and sampling***
  A search on four databases (PubMed, Cochrane, ScienceDirect, and Google Scholar) was conducted on 2 February 2022, to detect existing studies on the acceptability aspects related to the use of SAAgT. Search terms used were (self-administered tests OR self-testing OR self-collected OR at-home OR self-swabbing) AND (covid-19) AND (antigen) AND (acceptability OR knowledge OR perception OR capacity OR willingness OR social impact OR ethical impact OR health information system impact). The search included peer-reviewed articles published within the last five years regardless of country setting and population. Editorials, news briefs, communication briefs, and studies not written in English or Filipino were excluded from the search.
- ***Data analysis***
  This assessment applied content analysis to examine the retrieved relevant articles and identify common themes which were essential for the precoding systems. Open analysis was done to identify recurring themes and ideas from the collected studies. Since the purpose of the literature scoping was to identify possible preset codes, axial analysis, otherwise known as the second level of coding, was not conducted during this step.

### Focused group discussions

- ***Data collection and sampling***
  Four FGDs were conducted from 15–24 February 2022 by the HTA Philippines (HTA Council–Joint Subcommittee on Self-Administered Antigen Test and support staff from the HTA Division) via online platforms to reduce the risk of COVID-19 transmission. These included HCWs, at-risk groups, economic frontliners, and employers and managers of micro, small, and medium enterprises (MSMEs) and/or academic institutions. These groups were chosen because they were deemed to have diverse perspectives in health care which can be generalized to the wider context of acceptability in the local health system.
*FGD 1: HCWs—*Nine participants consisting of one barangay health worker and eight leaders of organizations representing physicians, pharmacists, nurses, caregivers, and dentists were included.
*FGD 2*: At-risk groups—Seven formal organizations for chronic illnesses, lung diseases, cancer, kidney transplantation, and HIV/AIDS were secured.
*FGD 3*: Economic frontliners—Six workers composed of a cab driver, a jeepney driver, delivery riders, a fieldworker, and a public school teacher were included in the FGD.
*FGD 4*: MSMEs and academic institutions—A total of seven representatives were included in the FGD, five of which are managers or administrative officers of an MSME in the field of human resources, air conditioning, pharmaceutical industry, and sugar industry, while there are two decision makers in their respective private schools.
  Due to the urgency of the assessment of SAAgT, snowball sampling was used to invite the participants. Some of the participants from previous FGDs conducted by HTA Philippines were invited and/or asked for referrals.
  Both Filipino and English were used as primary media of instruction for the first and last FGDs, while Filipino was the main medium for the second and third FGDs. Each FGD was facilitated by two to three representatives from the HTA Council. FGDs were conducted to have in-depth discussions and exchange of ideas and to observe the patterns of interaction among the participants [9].
- ***Data analysis***
  The preset codes were used to register the responses of the participants. All FGD responses were encoded using Google Docs.
  In order to analyze the raw data obtained from FGDs, both open and axial analyses were performed. For the open analysis, the codes were analyzed inductively and subsumed under preset codes that represent key decision points. New codes were included when responses did not fit any of the preset codes. The frequency of codes determined the number of times that participants mentioned or discussed the concept. If a code has been interpreted for at least three times in one FGD, then that code is considered as significant and is included in the analysis. Following the open analysis, axial analysis was performed by clustering first-level codes and recognizing patterns or relationships between the codes to generate themes from the users and implementers.
- ***Instrument***
  A semi-structured questionnaire was created by the HTA Philippines to guide the discussion of the participants’ perspectives, acceptability, and willingness to self-report. See Table 1 for the complete instrument used per FGD. Initial questions were added to capture the baseline knowledge and perception of the participants. The instrument also contained a brief context on SAAgT after every few questions to assess how the level of knowledge can affect their perception. The participants would then be asked whether they will change their previous answers given this new information. Context slides were not presented until after the sixth question. Members of the HTA Council validated the content of the questions and context slides based on the objectives of the FGDs. The tool was tailored to match the interest of subgroups with respect to COVID-19 testing.

**Table 1.** Questionnaire used in FGDs

FGD 1 questions
Can you share your experiences if you, your friends, or family needed COVID-19 testing? What characteristics must be present in a COVID-19 test to encourage testing? What have you heard about self-testing for COVID-19 infection? In your opinion, will doing self-administered antigen testing improve control of COVID-19 in the country? Do you think that DOH should recommend self-testing? In which situations will self-testing be useful? Why do you say so? Where do you think is the best distribution point for SAAgT kits? If you were given a SAAgT, would you use this? In what situations will you use it? What difficulties might you have? Follow-up: After watching the video, have your thoughts on self-testing kits changed? If yes, what changed and why? Will you recommend self-testing to your patients, family members, and friends? Why? If you tested POSITIVE, what would you do? What would you do if the result of your self-test is NEGATIVE? What if you have symptoms or you are exposed to a confirmed COVID-19 case? Do you think there should be a reporting mechanism for self-test results? If yes, how should this be reported? Any suggestions? Follow-up: After the presentation, will your action change after finding out the result?
FGD 2 questions
Maaari ba ninyong ibahagi ang karanasan ninyo o ng mga kakilala ninyo na nangailangan magpa-test para sa COVID-19? (refer to FGD 1, no. 1 for English translation) Anu-ano ang mga katangiang nais ninyo sa isang COVID-19 test upang makahikayat na magpa-test o gumamit ng testing? (refer to FGD 1, no. 2 for English translation) Ano na ang narinig ninyo tungkol sa self-antigen testing? (refer to FGD 1, no. 3 for English translation) Sa palagay ninyo, makakatulong ba ang paggamit ng self-antigen testing sa pagsugpo ng COVID-19 sa ating bansa? (refer to FGD 1, no. 4 for English translation) Dapat bang i-promote ng DOH ang self-antigen testing? (refer to FGD 1, no. 5 for English translation) Follow-up: Dapat bang magbigay ang gobyerno ng libreng self-antigen test kits? Para kanino at bakit? (Should the government provide free self-antigen test kits? For whom and why?) Sa palagay ninyo, saan pinakamadaling maipamimigay ang self-antigen test kits? (refer to FGD 1, no. 6 for English translation) Kung bibigyan kayo ng self-testing kit, gagamitin ba ninyo ito? Sa anong sitwasyon ninyo ito gagamitin? Ano kaya ang magiging problema sa paggamit nito? (refer to FGD 1, no. 7 for English translation) Follow up: Matapos panoorin ang video, nagbago ba ang pananaw ninyo tungkol sa mga self-test kit? Kung oo, ano pa ang iniisip ninyong posibleng balakid sa paggamit nito? (refer to FGD 1, no. 7, follow-up for English translation) Irerekomenda n’yo ba ang self-antigen testing sa inyong mga kapamilya at mga kaibigan? Bakit? (refer to FGD 1, no. 8 for English translation) Ano ang gagawin ninyo kung kayo ay nag-POSITIVE sa self-test kit? (refer to FGD 1, no. 9 for English translation) Ano kaya ang gagawin ninyo kung NEGATIVE ang resulta ninyo sa test? Paano kung may sintomas kayo o kung kayo ay na-expose sa isang taong positibo sa COVID-19? (refer to FGD 1, no. 10 for English translation) Sang-ayon ba kayo na i-report ang resulta ng inyong self-test? Bakit? Sa palagay ninyo saan o kanino mas karapat-dapat i-report ito? (Do you agree to report your self-test results? Why? Where or to whom do you think should these results be reported to?) Follow-up: Matapos ang presentation, magbabago ba ang gagawin ninyo pagkatapos malaman ang resulta ng test? (refer to FGD 1, no. 11, follow-up for English translation) Follow-up: May gusto pa ba kayong idagdag o imungkahi tungkol sa self-antigen testing? (Would you like to add anything further to the discussion on self-antigen testing?)
FGD 3 questions
Nakasubok na ba kayo o mga kakilala n’yo magpatest para sa COVID-19? (Have you or has anyone you know tried a COVID-19 test?) Sa tingin ninyo, alin sa mga katangiang ito ang pinakaimportante sa isang COVID-19 test? [Presyo ng test, Aksesibilidad, Bilis ng resulta, Tamang resulta, Iba pang katangian]. (Which of these characteristics do you think are most important in a COVID-19 test? [price of the test, accessibility, speed of results, accuracy of results, other characteristics) Ano na ang narinig ninyo tungkol sa self-antigen testing? (refer to FGD 1, no. 3 for English translation) Sa palagay ninyo, makakatulong ba ang paggamit ng self-antigen testing sa pagsugpo ng COVID-19 sa ating bansa? (refer to FGD 1, no. 4 for English translation) Dapat bang irekomenda ng gobyerno ang self-antigen test? Bakit? (refer to FGD 1, no. 5 for English translation) Sa palagay ninyo, saan pinakamainam maipamimigay ang self-antigen test kits? (refer to FGD 1, no. 6 for English translation) Kung bibigyan kayo ng self-testing kit, gagamitin ba ninyo ito? Sa anong sitwasyon ninyo ito gagamitin? Ano ang maaaring problema sa paggamit nito? (refer to FGD 1, no. 7 for English translation) Follow-up: Matapos panoorin ang video, nagbago ba ang pananaw n’yo sa paggamit ng self-antigen test? (refer to FGD 1, no. 7, follow-up for English translation) Irerekomenda mo ba ang self-antigen test sa inyong mga kapamilya at mga kaibigan? Bakit? (refer to FGD 1, no. 8 for English translation) Ano ang agam-agam mo kung mag-POSITIVE ang resulta mo sa self-antigen test? (refer to FGD 1, no. 9 for English translation) Kung NEGATIVE naman ang resulta mo, ano ang gagawin mo? Paano kung may sintomas kayo o kung kayo ay na-expose sa isang taong positibo sa COVID-19? (refer to FGD 1, no. 10 for English translation) Sang-ayon ka ba na dapat i-report ng mga tao ang resulta ng kanilang self-antigen test (positibo o negatibo man)? (Do you agree to report your self-test results, regardless if positive or negative?) Follow up: Ano ang pananaw mo ngayon tungkol sa mga gagawin pag nalaman ang mga resulta? (What is your perspective about actions to take upon knowing your results?) Follow-up: May gusto pa kayong idagdag o imungkahi tungkol sa self-antigen testing? (Would you like to add anything further to the discussion on self-antigen testing?)
FGD 4 questions
Can you share your experiences, if you or any of your friends or family underwent COVID-19 testing? Follow-up: As business owners or managers, or academic managers, do you do any kind of COVID-19 testing for your employees? If you were to choose, what characteristics in a COVID-19 test kit would you be looking for? What have you heard about self-antigen testing for COVID-19 infection? In your opinion, how useful do you think would self-antigen testing be in improving the control of COVID-19 in the country? How about its impact in the day-to-day operations of your business/school? Do you think that DOH should promote self-antigen testing? Follow-up: Should the government provide free self-antigen test kits? For whom and why? Where is the best distribution point for the self-antigen testing kits? Would you be willing to use self-antigen testing for your employees? If yes, how often should testing be done and until when? In what situations will you use it? Would you be willing to shoulder the cost of the self-test kits? What problems do you foresee in the use of self-antigen test? Follow-up: After watching the video, have your thoughts on the self-antigen testing kit changed? If yes, what changed and why? Will you recommend self-antigen testing to your employees, family members, and friends? Why? If your employee tested POSITIVE, what would you do? What would you do if the result of your employee’s test is NEGATIVE? What would you do if your employee has symptoms or has been exposed? Follow-up: If your employee has been exposed to a COVID case, would you use self-antigen testing for the purpose of determining if they could: [1] stay in the workplace (or in the school, as applicable); or [2] return to work/ school? How open would you be to the use of self-antigen test kit for that purpose? Do you agree that results of self-antigen test of your employees (whether positive or negative) should be reported? Why or why not? In your opinion, where or to whom should this be reported? Follow-up: After the presentation, will your previously stated perceptions and attitudes change after knowing the test results of your employees? If yes, why? Follow-up: Do you have anything to add or suggest about self-antigen testing for COVID?

FGD, focus group discussion.

### Data privacy and ethical considerations

Disclosure of conflict of interest and signed consent forms for FGD participation and FGD session recording were obtained from the participants prior to the start of each session. The actual names of the participants were anonymized. For the FGDs on HCWs and at-risk groups, the names of the organization were used in lieu of the actual participant names throughout the report.

Pursuant to the Data Privacy Act in the Philippines [10], access to all personal information of the participants can only be disclosed to authorized personnel from the HTA Philippines or as formally requested in written form by any recognized judicial court in the Philippines.

For all FGDs, ethical considerations on confidentiality and the right to information were strictly observed. However, the HTA Philippines recognizes that participants may choose to disclose their personal information outside of the FGDs; in which case, the HTA Philippines does not have control over it.

### Scope and limitations

Since the sampling of participants was done through snowballing, the representation from the FGDs may be limited or not exhaustive of the characteristics of the target key stakeholders. Due to the risk of transmission of COVID-19, the organizers were only able to conduct a virtual FGD instead of a face-to-face discussion. As such, this assessment may have excluded the part of the population that do not have access to or are not adept with high technology devices.

## Results and discussion

The systematic search of studies for preset codes yielded 2,657 results. After full-text screening, seventeen articles were included as the basis for precoding. A total of twenty-eight preset codes were extracted from literature review, as seen in Table 2. Apart from the preset codes, twelve new first-level codes were added across all FGDs after thematic analysis. These first-level codes have been further saturated into seven themes, which are divided into converging and diverging themes.

**Table 2.** First-level codes

Preset codes	
Alternative routes for information, education, and communication (IEC) Alternative routes for specimen collection Autonomy on testing Community mobilization in self-testing Confidence in the accuracy of results Confidence in the healthcare system Conformity and self-testing Consequences of isolation (economic) Disability and self-testing Discomfort in self-administration of test Educational attainment as predictor of compliance Fear of being discriminated Gender-based preferences in self-testing Incurred cost by the institution as perceived barrier	Ineffective health education for COVID-19 Out-of-pocket costs as barrier to testing Perceived vulnerability to COVID-19 Positive reinforcement for testing Prevention of cross-infection Proximity of testing Rapid release of results Risk-based testing Self-efficacy and self-testing Sociocultural differences in testing acceptability Streamlining self-testing Testing and case management Testing for workload reduction in health facilities (HFs) Time convenience of testing
FGD 1	
*Old codes (available in preset codes)* Alternative routes for IEC Alternative routes for specimen collection Autonomy on testing Community mobilization in self-testing Confidence in the accuracy of results Confidence in the healthcare system Discomfort in self-administration of test Fear of being discriminated Incurred cost by institutions as perceived barrier Ineffective health education for COVID-19 Out-of-pocket costs as barrier to testing Perceived vulnerability to COVID-19 Prevention of cross-infection Proximity of testing Rapid release of results Risk-based testing Self-efficacy and self-testing Testing and case management	*New codes* Centralized reporting and patient information system Confidence in regulation of test kits Hiya as a barrier to COVID-testing Human resource capacity building in SAAgT
FGD 2	
*Old codes (available in preset codes)* Alternative routes for IEC Autonomy in testing Community mobilization in self-testing Confidence in the accuracy of results Confidence in the healthcare system Disability and COVID testing Discomfort in administration of self-test Fear of being discriminated Ineffective health education for COVID-19 Isolation as a consequence of testing Out-of-pocket costs as barrier to testing Perceived vulnerability to COVID-19 Positive reinforcement for testing Prevention of cross-infection Proximity of testing Rapid release of results Risk-based testing Streamlining self-testing Testing and case management Time convenience of testing	*New codes* Designated access point for test kits Centralized reporting and patient information system Hiya as a barrier to COVID-testing Padrino System
FGD 3	
*Old codes (available in preset codes)* Alternative routes for IEC Community mobilization in self-testing Confidence in the accuracy of results Fear of being discriminated Ineffective health education for COVID-19 Isolation as a consequence of testing Out-of-pocket costs as barrier to testing Perceived vulnerability to COVID-19 Prevention of cross-infection Rapid release of results Risk-based testing Self-efficacy and self-testing Streamlining self-testing Testing and case management	*New codes* Designated access point for test kits Centralized reporting and patient information system Grapevine communication Hiya as a barrier to COVID testing Perceived superiority of HCP administered test over SAAgT
FGD 4	
*Old codes (available in preset codes)* Alternative routes for IEC Alternative routes for specimen collection Autonomy in testing Confidence in the accuracy of results Confidence in the healthcare system Ineffective health education for COVID-19 Isolation as a consequence of testing Out-of-pocket costs as barrier to testing Perceived vulnerability to COVID-19 Positive reinforcement for testing Prevention of cross-infection Rapid release of results Risk-based testing Sociocultural differences in testing acceptability Streamlining self-testing Testing and case management Time convenience of testing	*New codes* Designated access point for test kits Centralized reporting and patient information system Employer-supported testing Full disclosure of self-test results Increased access to GIDAs for self-testing Padrino System Storage and stability of COVID-19 tests

### Converging themes

Four converging themes were classified in terms of three categories: general view of testing, programmatic considerations for SAAgT, and preferred test characteristics. The converging themes are summarized in Table 3.

**Table 3.** Converging and diverging themes

Converging themes	Diverging themes
Hiya as a barrier to COVID-19 testing Community mobilization on self-testing Streamlining self-testing Personal convenience as a motivator to testing	Padrino System Perceived financial barriers in testing Increased access for rural areas and GIDAs

GIDAs, geographically isolated and disadvantaged areas.

#### General view of testing

##### Theme 1: Hiya as a barrier to COVID-19 testing

*Hiya*, in *Sikolohiyang Pilipino* (Filipino Psychology), elicits many different meanings depending on the affixes attached to the root word. In the context of COVID-19, this refers to participants’ feeling of embarrassment or sense of propriety in letting others know that they have tested positive for COVID-19 due to the possibility of being stigmatized or becoming the “talk of town” [11]. Because of *hiya*, participants may choose to hide their results or forgo testing.

##### Hiya in acquiring testing services

*Hiya* is seen as a disadvantage because this can make individuals complacent about isolation and quarantine protocols. Some participants from the at-risk groups highlighted unique experiences of *hiya* in the context of testing for other conditions or illnesses. One such example is the discrimination experienced by patient groups whenever they attempt to get testing services. A participant shared that neurofibromatosis patients faced difficulty in getting tested in health facilities due to being discriminated upon for having visible tumors.

##### Hiya in reporting positive results

Reporting positive results was seen as a source of stigma in communities. Participants from the at-risk and economic frontliner groups expressed concerns over having “astronaut-like” authorities enter their homes and “forcefully” take COVID-positive individuals into quarantine facilities, often against their will. These said authorities would also reportedly coerce the families of COVID-positive individuals to isolate themselves in their own homes. Other members of the community tend to avoid these COVID-positive individuals. Two market vendors repeatedly emphasized their fear of being gossiped about as COVID-positive. This image of being "contagious” may become an individual’s persona even if the actual infection has been resolved, that being COVID-positive has already become a badge of carriage.

#### Programmatic considerations for SAAgT

##### Theme 2: Community mobilization on self-testing

Participants from all groups agreed that a community-centered, grassroots-based approach must be implemented to increase the salience of the people for testing with SAAgT. Participants expressed that individuals may have different perceptions about their vulnerability to COVID-19, which, in turn, affect their motivation to undergo testing. People who perceive themselves as not vulnerable to COVID-19 would not be willing to undergo SAAgT and vice versa. This, coupled with misconceptions about testing, SAAgT, and COVID-19, can potentially decrease the utilization of SAAgT. As such, participants from all FGDs agreed that communities, and not just individuals, must be mobilized to take an active role in testing implementation. Suggested steps include alternative routes to information, education, and communication platforms and positive forms of reinforcement to increase the confidence of individuals toward SAAgT.

Participants enumerated specific topics such as test procurement, test use, test interpretation, and test kit disposal, taught through social media, lectures, training, or handouts which are easy to understand. They further explained how other methods can improve IEC campaign reach in the community, such as instructions in the vernacular language of the target audience, return demonstrations, and video presentations. Some participants complained about the instructions on currently used test kits because these are written in non-native languages (e.g., Chinese) and may not be readily understandable to an average Filipino. The self-test kit inserts should be stated in a language that is spoken by the average person in a particular area or region (e.g., Tagalog for Tagalog speakers, Bisaya among Visayans).

When incentives are given for individuals in the community, positive attitudes toward SAAgT are more likely to be reinforced, resulting in higher utilization. These incentives that can reinforce behavior are not only limited to the usual “*ayuda*” or government dole outs in the form of monetary and nonmonetary aid. One participant cautions about the misuse of incentives for fostering trust in SAAgT. According to her, people from the communities do not only need materialistic incentives but also need explanations on the testing processes and reassurance that they “would not be taken away from their families” as a consequence of isolation. Incentives, in this sense, are not limited to materialistic rewards but can also include adding official work leaves on top of sick leaves to accommodate testing, verbal appreciation, and reassurance for performing testing, among others.

##### Theme 3: Streamlining self-testing

Adopting the use of SAAgT into currently existing policies and processes for testing strategies can make the testing initiative relatively easier to implement.

##### Distribution of self-test kits

Using health facilities within the national service delivery network as a designated access point will allow for better reach of self-test kits, especially among GIDAs. Participants from the HCW sector emphasized that test kits should be controlled by regulatory agencies in order to ensure their quality and avoid unregulated distribution. This could prevent the risk of buying counterfeit test kits from online stores as well as possible contamination and issues of faulty handling. A participant suggested that SAAgTs should be delivered from *barangay* to the houses of those at risk for COVID-19, those needing the kits as a requirement for work-related purposes, or for case management. Participants expressed concerns about having to line up to retrieve them, supposedly similar to the experiences of the people with “vaccine rollouts.” In this system, participants believe that priority in the distribution should be those exposed to suspected, probable, or active COVID-19 case, vulnerable sectors (e.g., older adults, PWDs, people with comorbidities), and indigent individuals. Moreover, the at-risk groups recognized the limitations in the government resources that could be allocated for self-test kits. They expounded that only self-test kits in excess of those given to the three groups should be given to individuals in the formal economy because they have the capacity to pay for test kits.

##### Reporting of self-test results

The participants expressed the need for centralized reporting and systematic patient information systems to make the reporting of health status more convenient. The national government can use this centralized system for surveillance and screening of COVID-19. It also has a role in the appropriate referral of individuals who test positive for COVID-19. A participant noted that in cases or areas where consultation with an actual physician is not accessible or feasible, the people who are managing this centralized reporting and patient information system can give advice regarding teleconsultation. One participant in the economic frontliner group also suggested using applications or websites for case reporting. While many areas in the Philippines have access to internet or data signal, rural areas and GIDAs may not have adequate internet signal. Strengthening internet connectivity among these areas will thus make a centralized database for patient information and reporting system more feasible.

Participants from the MSME group further expressed the need for a national issuance on COVID-19 testing in the workplace, including detailed guidelines for self-testing. They suggested that such guidelines should be considerate toward employees who do not have leave benefits, because otherwise employees will be discouraged to report their results honestly for fear of salary deductions or other forms of sanctions from being absent. Employees may be encouraged to undergo self-testing if employers allow work from home, provide paid leaves, provide spaces for quarantine, and shoulder the cost of the testing itself (including subsequent tests). These various employer-support mechanisms serve as incentives for their constituents to comply with self-testing and honest reporting.

#### Preferred test characteristics

##### Theme 4: Personal convenience as a motivator to testing

Personal convenience was believed to positively influence the perception and utilization of self-testing. This revolves around the characteristics of SAAgT itself such as quality, affordability, proximity, and time convenience. Stakeholders want self-testing to possess certain attributes that can increase the desirability of self-testing.

Subsidizing SAAgT can help avoid lengthy times for waiting or traveling toward health facilities, as compared to RT-PCR and RAgT. HCWs and at-risk groups explained that self-testing at home can be more convenient for individuals who will be needing testing because it lessens the “grueling” time of lining up or waiting for RT-PCR or RAgT and the long turnaround time for the results to be released. Individuals at high risk for severe COVID-19 especially cannot go to testing facilities during times of surge because they want to avoid crowded areas as much as possible. They also lean toward choosing the option which delivers faster test results especially when it will be used as a requirement in the workplace, for travel, or before undergoing certain hospital-based treatments (that is dialysis) or procedures (that is surgery).

Target users also seek forms of self-testing which are easy to use, less invasive, and involve the least amount of discomfort. Some participants suggested test kits that use alternative routes of specimen collection (e.g., saliva testing).

The decreased costs associated with self-testing is another important characteristic commonly raised by all FGD groups. This includes transportation costs, logistical costs (storage), and, most importantly, the cost of the kits themselves compared with other forms of testing. For economic frontliners such as delivery riders with unstable income, they desire testing that will limit or even eliminate loss of income and productivity time. Some participants verbalized that they prefer if the self-testing be free or at least subsidized by the government.

### Diverging themes

As the FGD groups comprise individuals with different backgrounds and experiences which result in diversifying beliefs and perspectives, differences were expected among FGD groups in their acceptability of SAAgT. There are three diverging themes classified into the three aforementioned categories, as summarized in Table 3.

#### General view of testing

##### Theme 5: Padrino system

The *Padrino System*, otherwise known as the *Palakasan System*, is a value system in Filipino culture in which a person gains favor because of close ties or relationships with a supervisor/official, familial affiliations, or “*utang na loob*” [debt of gratitude] of the one giving the favor to the one gaining the favor [12]. This theme was highlighted by the at-risk and MSME groups. They raised the possibility of uneven distribution of SAAgT or being given free to only the favored because of their first-hand observation with LGU-level or corporate-level red taping, particularly prioritization of those with connections for RT-PCR testing. Participants expressed fears that access can be a problem for COVID-19 testing because some public officials may distribute the kits to their allies. This system decreases the perceived utility of potential COVID tests among target individuals and consequently decreases COVID test utilization.

#### Experiences/barriers in testing

##### Theme 6: Perceived financial barriers in testing

Among participants in the HCW and MSME groups, the low cost of testing is perceived only as a preferred quality of test—that it is more of a “want,” rather than an actual need. For economic frontliners and at-risk participants, however, the cost of testing can take a significant portion of their earnings or revenue that would otherwise be used for more necessary expenses (e.g., food, tuition, rent, etc.). Some participants in this group emphasized that the cost of testing makes them hesitant to get tested. One participant even exclaimed that individuals who are “no work, no pay” must be prioritized for free testing, if this were to be provided. One market vendor also suggested making testing free for market vendors because they are constantly exposed to other people. A jeepney driver expressed that testing ought to be given for free to drivers because they encounter multiple passengers per day in their line of work, which would mean more exposure to people with unknown COVID-19 status. Overall, participants agreed that free or subsidized test kits must prioritize the financially needy and vulnerable populations. If the government is unable to provide it for free, cost burden may potentially be alleviated through subsidization programs or as part of private health insurance policies.

#### Programmatic considerations for SAAgT

##### Theme 7: Increased access for rural areas and GIDAs

One of the programmatic considerations for SAAgT is the need to plan carefully the provision of self-testing to rural areas and GIDAs. This includes concerns about population characteristics and logistical concerns (e.g., storage, infrastructure). They explained their sentiment that their staff residing in areas in Visayas and Mindanao might not have the same level of access to self-test and its adjunct services compared to those living in Metro Manila. These adjunct services include the system of reporting results, the distribution points, and the education and guidance, which are all more accessible in urban areas. Individuals in rural areas such as farmers may not necessarily have the same level of willingness to do testing like an individual who lives in highly urbanized areas. The limited information reach in testing among rural areas and GIDAs as well as inaccessibility to healthcare facilities and services potentially result in noncompliance to testing guidelines and lack of salience in SAAgT among community members.

Another aspect of increasing access to testing is the considerations about the storage and stability of COVID-19 tests. For instance, a school owner said that SAAgT is preferred because it requires less complicated storage facilities compared to RT-PCR. In order to ensure SAAgT’s efficacy, coordinating with “drugstores” was suggested to help in maintaining the required conditions for storage and to maintain kit stability. If test kits will be distributed in rural areas and GIDAs, where infrastructure is poor or insufficient, developing strategic storage systems in these areas should be among the key priorities to consider.

### Policy recommendation

This assessment lists policy recommendations which may potentially improve the acceptability of SAAgT, increase confidence in using SAAgT, and increase willingness to report results. These recommendations for using SAAgT based on the aforementioned research questions and results can be found in Table 4.

**Table 4.** Policy recommendations for SAAgT use in the Philippines

Policy objectives	Policy recommendations
To increase the level of acceptability in using SAAgT	Increase IEC campaigns about SAAgT across communities This can reduce negative perceptions and fear about COVID-19 and using COVID-related health services (i.e., screening) by letting constituents understand that COVID-19 is a preventable and manageable condition, thus promoting nondiscrimination in communities. This can help constituents understand the importance of COVID-19 testing and how SAAgT can be a convenient alternative to conventional facility-based testing.
To promote SAAgT use by increasing accessibility to SAAgT	Create policies which establish the availability of SAAgT in common access points such as hospitals, health centers, pharmacies, and convenience stores. Create policies which enable subsidization of SAAgT, including additional financial protection for individuals living in poverty (e.g., make test kits free for eligible populations).
To increase capability in self-administering SAAgT	Create easy-to-understand IEC materials which are readily available online or when procuring SAAgT from access points. Mandate designated personnel in all levels of health facilities to teach service users about the use of SAAgT, including live demonstrations.
To increase voluntary reporting after obtaining results from SAAgT	This is correlated with acceptability in the use of SAAgT. IEC campaigns can also be used to help constituents understand the importance of honestly reporting results and the gravity of the consequences of doing otherwise. Moreover, a community free from stigma and discrimination will likely enable honest reporting. Mandate surveillance centers to include SAAgT results in official COVID-19 case count databases. Concurrently, create policies mandating the reporting of results which turn out positive.

SAAgT, self-administered antigen test.

## Conclusion

All FGD groups expressed favorable reviews on the implementation of SAAgT in public health care because it can potentially reduce the burden of health facility-administered tests in terms of time convenience, affordability, and prevention of cross-infection. They further noted that some potential issues to SAAgT implementation must be resolved to ensure its efficient rollout. Cost, geography (GIDA), discomfort, lack of confidence in its accuracy, and lack of knowledge in using the test are some of the barriers for implementation. Moreover, many participants expressed their unwillingness to report self-test results due to fear of stigma and loss of livelihood as a result of forced isolation. To effectively utilize and reap the benefits of SAAgT, implementation must include prioritization for free/subsidized kits, a designated access point and reporting system, and an IEC campaign. The HTA Council recommendation considered in crafting implementing guidelines the use of SAAgT (i) for diagnosis of suspect or probable cases in people at high risk for COVID-19 and (ii) for screening and diagnosis of HCWs.
